# Functional Promoter -31G>C Variant in Survivin Gene Is Associated with Risk and Progression of Renal Cell Cancer in a Chinese Population

**DOI:** 10.1371/journal.pone.0028829

**Published:** 2012-01-25

**Authors:** Chao Qin, Qiang Cao, Pu Li, Xiaobing Ju, Meilin Wang, Jiawei Chen, Yilong Wu, Xiaoxin Meng, Jian Zhu, Zhengdong Zhang, Qiang Lu, Changjun Yin

**Affiliations:** 1 Department of Urology, The First Affiliated Hospital of Nanjing Medical University, Nanjing, China; 2 Department of Molecular and Genetic Toxicology, School of Public Health, Nanjing Medical University, Nanjing, China; Ohio State University Medical Center, United States of America

## Abstract

**Background:**

Survivin is an inhibitor of apoptosis protein and is involved in the occurrence and progression of human malignancies. Recently, a functional polymorphism (−31G>C, rs9904341) in the promoter of *survivin* has been shown to influence its expression and confer susceptibility to different types of cancer. The present study was aimed to investigate whether the polymorphism also influences susceptibility and progression of renal cell cancer (RCC) in a Chinese population.

**Methods:**

We genotyped this polymorphism using the TaqMan assay in a case-control study comprised of 710 RCC patients and 760 controls. The logistic regression was used to assess the genetic association with occurrence and progression of RCC.

**Results:**

Compared with the genotypes containing G allele (GG and GC), we found a statistically significant increased occurrence of RCC associated with the CC genotype [*P* = 0.006, adjusted odds ratio (OR) = 1.38, 95% confidence interval (CI) = 1.08–1.76]. The polymorphism was associated with risk of developing advanced stage (OR = 2.02, 95%CI = 1.34–3.07) and moderately differentiated (OR = 1.75; 95%CI = 1.20–2.54) RCC. Furthermore, the patients carrying the CC genotype had a significantly greater prevalence of high clinical stage disease (*P_trend_* = 0.003). Similar results were also observed when we restricted the analysis to clear cell RCC, a major histological type of RCC.

**Conclusions:**

Our results suggest that the functional −31G>C polymorphism in the promoter of *survivin* may influence the susceptibility and progression of RCC in the Chinese population. Large population-based prospective studies are required to validate our findings.

## Introduction

Renal cell cancer (RCC) accounts for more than 90% of all renal malignancies [1]. The incidence rates of RCC vary among different populations, with higher rates in Europeans and lower rates in Asians [1]. The exact causes of RCC are poorly understood. To date, only a few risk factors for RCC have been established, including cigarette smoking, obesity, hypertension and diabetes [1]. Renal tumorigenesis is a complex process determined by the interactions between environmental and genetic factors [2]. The identification of genetic susceptibility of RCC, although is still needed further investigation, has enlightened the molecular pathogenesis of this disease [1], [3], [4], [5]. It also has been suggested that genetic variation in candidate-genes influenced the progression and prognosis of RCC [6], [7], [8], [9].

Dysregulation of apoptosis has been implicated in carcinogenesis through abnormally prolonging cell survival and facilitating the accumulation of transforming mutations[10]. Survivin is a structurally unique member of the inhibitor of apoptosis protein family that suppresses apoptosis and regulates cell division [11], [12]. Over-expression of survivin was frequently observed in a variety of human malignancies [11], such as colorectal cancer [13], lung cancer [14], hepatocelluar carcinoma [15], pancreatic cancer [16], and osteosarcoma [17]. Besides, over-expression of survivin is correlated with poor prognosis of these cancers. It has been demonstrated that sufficient expression of survivin messenger RNA and protein were detected in RCC cell lines but not in normal human kidney epithelial cell line [18]. Elevated expression of survivin was also observed in RCC tissues compared with adjacent normal tissues [18], [19]. For the outcome of RCC patients, over-expression of survivin was significantly associated with advanced tumor stage, tumor grade and lymph node metastasis [19], [20]. Besides, RCC patients with high survivin levels had a significantly shorter overall survival time than those with low levels [21], [22].

Recently, a −31G>C (rs9904341) single-nucleotide polymorphism, which is located at the cell cycle-dependent elements (CDE) and cell cycle homology regions (CHR) repressor binding site of the *survivin* promoter, has been demonstrated to influence *survivin* expression by modifying the binding affinity of the CDE/CHR repressor [23], [24]. The −31G allele was shown to have a significantly lower transcriptional activity than the −31C allele [25]. To date, several epidemiological studies have suggested that this polymorphism was associated with the risk and/or prognosis of various carcinomas [26]. However, the role of this polymorphism in the etiology of RCC has never been specifically investigated before. Considering the crucial role that survivin plays in the occurrence and progression of RCC, we hypothesized that the functional −31G>C polymorphism in the promoter of *survivin* could be a potentially genetic marker to predict RCC risk and progression. To test this hypothesis, we genotyped this polymorphism in a case-control study comprised of 710 cases and 760 controls in a Chinese population and assessed the genetic association with occurrence and progression of RCC using logistic regression analysis.

## Materials and Methods

### Ethics Statement

The study was approved by the Institutional Review Board of the Nanjing Medical University, Nanjing, China. At recruitment, written informed consent was obtained from all participants involved in this study.

### Study population

This is an ongoing molecular epidemiologic study of RCC conducted in the First Affiliated Hospital of Nanjing Medical University, Nanjing, China, from May 2004. The design of the study and the inclusion criteria of the subjects were previously described elsewhere [6]. In brief, all subjects in our study are ethnic Han Chinese coming from different families and have no blood relationship. All the patients were newly diagnosed with histopathologically confirmed, incident RCC without prior history of other cancers or previous chemotherapy or radiotherapy, and were consecutively recruited without restriction of age and sex. Disease was classified according to World Health Organization criteria and staged according to the American Joint Committee on Cancer TNM classification. The Fuhrman scale was used to assess tumor nuclear grade. Histological sections of all cases were reviewed by two pathologists independently. The controls were recruited from healthy subjects who were seeking physical examination in the outpatient departments at the hospital and were frequency-matched to the cases on age (±5 years) and sex. Hypertension was defined as systolic BP (SBP)≥140 mmHg and/or diastolic BP (DBP)≥90 mmHg, and/or being on drug therapy. Diabetes diagnosis was measured as self reported earlier diagnosis, fasting blood glucose ≥6.1 mmol/l, and/or treatment for earlier diagnosed diabetes mellitus. Compared to our previously published studies [6], a further 90 cases and 137 controls recruited recently were added to the present study.

### DNA extraction and polymorphism genotyping

Total genomic DNA was extracted from the peripheral blood by proteinase K digestion and phenol–chloroform extraction. The genotyping of the −31G>C polymorphism (rs9904341) was carried out using predesigned TaqMan SNP Genotyping Assays (Applied Biosystems, Foster City, CA, USA). The primer and probe sequences are summarized in Table S1. Amplification was performed under the following conditions: 50°C for 2 min, 95°C for 10 min followed by 45 cycles of 95°C for 15 sec, and 60°C for 1 min. According to the manufacturer's instructions, amplifications and analysis were carried out in the 384-well ABI 7900HT Real-Time PCR System (Applied Biosystems) and the SDS 2.4 software were used for allelic discrimination. For quality control, four negative controls were included in each plate and 5% of the samples were randomly selected for repeated genotyping for confirmation; and the results were 100% concordant.

### Statistical analysis

The differences in categorical variables such as gender, smoking status, drinking status, and in the frequency distribution of −31G>C alleles and genotypes between the cases and controls were evaluated by Pearson's chi-square test. The differences in continuous variables such as age and BMI were tested by Student's t-test. Before analysis, allele frequencies of the −31G>C polymorphism in the controls was tested against departure from the Hardy-Weinberg Equilibrium (HWE) using the Goodness-of-fit Chi-square test. Associations between the −31G>C polymorphism and RCC risk were evaluated by computing odds ratios (ORs) and 95% confidence intervals (CIs) from unconditional logistic regression analysis with the adjustment for possible confounders. A two-side *P* value of less than 0.05 was considered as statistically significant. All the analyses were carried out with the software SAS 9.1.3 (SAS Institute, Cary, NC, USA).

## Results

The selected characteristics between the RCC patients and control subjects are presented in Table 1. The cases and controls appeared to be well matched on age and sex (*P* = 0.753 and 0.832, respectively). Moreover, no significant differences were observed between the cases and controls with regards to BMI and drinking status (*P* = 0.078 and *P* = 0.120, respectively). However, more smokers, hypertension patients and diabetics were presented in the cases compared with controls (*P* = 0.035, <0.001 and <0.001, respectively). The majority of patients (84.8%) had the conventional clear cell cancer. When stratified according to the clinical stage, 62.8%, 19.6%, 7.3% and 10.3% of the patients had stage I, II, III and IV disease, respectively. The percent of nuclear grade from I to IV was 19.2%, 48.0%, 24.5%, and 8.3%, respectively.

**Table 1:** Distribution of selected variables between the renal cell cancer cases and the control subjects.

Variables	Cases (n = 710)		Controls (n = 760)		*P* *
	N	%	N	%	
Age (mean ± SD), years	56.9±11.9		56.8±11.6		0.753
≤57	364	51.3	423	55.7	0.092
>57	346	48.7	337	44.3	
BMI (mean ± SD), kg/m^2^	24.1±2.8		23.8±3.2		0.078
<24	346	48.7	391	51.5	0.298
≥24	364	51.3	369	48.5	
Sex					
Male	454	63.9	490	64.5	0.832
Female	256	36.1	270	35.5	
Smoking status					
Never	444	62.5	515	67.8	0.035
Ever	266	37.5	245	32.2	
Drinking status					
Never	508	71.6	571	75.1	0.120
Ever	202	28.5	189	24.9	
Hypertension					
No	444	62.5	444	73.0	<0.001
Yes	266	37.5	205	27.0	
Diabetes					
No	611	86.1	716	94.2	<0.001
Yes	99	13.9	44	5.8	
Clinical stage					
I	446	62.8			
II	139	19.6			
III	52	7.3			
IV	73	10.3			
Grade					
I	136	19.2			
II	341	48.0			
III	174	24.5			
IV	59	8.3			
Histology					
Clear cell	602	84.8			
Papillary	22	3.1			
Chromophobe	39	5.5			
Unclassified	47	6.6			

*Student's t-test for age and BMI distributions between cases and controls; two-sided χ2 test for other selected variables between cases and controls.

The genotype and allele frequencies of the −31G>C polymorphism are shown in Table 2. The genotype frequencies in controls and patients both conformed to HWE (*P* = 0.467 and 0.610, respectively). The frequencies of the GG, GC and CC genotypes among cases were significantly different from those among controls (*P* = 0.015). The difference in the frequencies distribution of G and C allele among case and controls was also significant (*P* = 0.006). Compared with individuals carrying G allele (GG/GC), individual with CC genotype had a significantly increased susceptibility to RCC occurrence (OR = 1.38, 95%CI = 1.08–1.76, *P* = 0.006). Furthermore, the variant −31C allele was also associated with an increased risk for RCC, compared with the −31G allele (OR = 1.11, 95%CI = 1.03–1.20, *P* = 0.006). Similar results were observed when we restricted the analysis to clear cell RCC (Table 2). These results suggested that the *survivin* −31G>C polymorphism had effect on RCC occurrence.

**Table 2:** Genotype and allele frequencies of *survivin* −31G>C polymorphism among the cases and controls and the associations with risk of renal cell cancer.

Genotypes	Controls, n (%)	Renal cell cancer patients			Clear cell renal cell cancer patients		
		N (%)	*P* *	OR (95% CI)*	n (%)	*P* *	OR (95% CI)*
−31G>C							
GG	215 (28.3)	172 (24.2)	0.008	1.00 (reference)	147 (24.4)	0.022	1.00 (reference)
GC	385 (50.7)	345 (48.6)		1.14 (0.89–1.47)	295 (49.0)		1.14 (0.88–1.49)
CC	160 (21.0)	193 (27.2)		1.50 (1.12–2.02)	160 (26.6)		1.42 (1.07–1.98)
*P*_trend_				0.006			
GG+GC	600 (79.0)	517 (72.8)	0.011	1.00 (reference)	443 (73.4)	0.032	1.00 (reference)
CC	160 (21.0)	193 (27.2)		1.38 (1.08–1.76)	159 (26.4)		1.32 (1.02–1.71)
G allele	815 (53.6)	689 (48.5)	0.006	1.00 (reference)	590 (49.0)	0.017	1.00 (reference)
C allele	705 (46.4)	731 (51.5)		1.23 (1.06–1.42)	614 (51.0)		1.20 (1.03–1.40)

*Adjusted for age, sex, BMI, smoking status, drinking status, diabetes and hypertension in logistic regression model. CI, confidence interval; OR, odds ratio.

We then evaluated the effect of the polymorphism on RCC risk stratified by age, BMI, sex, smoking status, smoking level and drinking status. The results were summarized in Table S2. Although the increased risk appeared to be more evident in the subgroups of young subjects, females, heavy smokers and ever drinkers, no significant heterogeneity between the subgroups was observed which implicated independent genetic effects (all *P*_heterogeneity_>0.05). We then examined the effects of the polymorphism on the progression of RCC. As presented in Table 3, the −31CC genotype was associated with risk of developing advanced stage RCC (OR = 2.02, 95%CI = 1.34–3.07) and moderately-differentiated RCC (OR = 1.75; 95%CI = 1.20–2.54). As shown in Table 4, in the case group, patients carrying the CC genotype had a significantly greater prevalence of high clinical stage disease (*P* = 0.021 for RCC and *P* = 0.018 for ccRCC) in a dose–response manner (*P*_trend_ = 0.003). However, no association between this polymorphism and tumor grade in cases was observed (*P* = 0.379 for RCC, *P* = 0.700 for ccRCC).

**Table 3:** The associations between *survivin* −31G>C polymorphism and the progression of renal cell cancer.

Categories	Renal cell cancer patients				Clear cell renal cell cancer patients			
	CG+GG	CC	*P* *	OR (95% CI)*	CG+GG	CC	*P* *	OR (95% CI)*
Controls (n = 760)	600 (79.0)	160 (21.0)	0.011	1.38 (1.08–1.76)	600 (79.0)	160 (21.0)	0.032	1.32 (1.02–1.71)
Cases (n = 710)								
Clinical stage								
Localized (I+II)	437 (74.7)	148 (25.3)	0.096	1.25 (0.96–1.62)	388 (75.2)	128 (24.8)	0.145	1.22 (0.93–1.60)
Advanced (III+IV)	80 (64.0)	45 (36.0)	0.001	2.02 (1.34–3.07)	54 (62.8)	32 (37.2)	0.001	2.20 (1.36–3.56)
Grade								
Well-differentiated (I+II)	354 (74.2)	123 (25.8)	0.088	1.27 (0.97–1.67)	319 (74.7)	108 (25.3)	0.119	1.25 (0.94–1.67)
Moderately-differentiated (III)	118 (67.8)	56 (32.2)	0.004	1.75 (1.20–2.54)	98 (70.0)	42 (30.0)	0.030	1.58 (1.05–2.39)
Poorly-differentiated (IV)	45 (76.3)	14 (23.7)	0.649	1.16 (0.61–2.19)	25 (71.4)	10 (28.6)	0.269	1.54 (0.72–3.30)

*Adjusted for age, sex, BMI, smoking status, drinking status, diabetes and hypertension in logistic regression model. CI, confidence interval; OR, odds ratio.

**Table 4:** The associations between *survivin* −31G>C polymorphism and clinical characteristics of renal cell cancer patients.

Categories	Renal cell cancer patients				Clear cell renal cell cancer patients			
	CG+GG	CC	*P* *	OR (95% CI)*	CG+GG	CC	*P* *	OR (95% CI)*
Clinical Stage								
I	342 (66.1)	104 (53.9)		1.00 (reference)	305 (69.0)	89 (55.6)		1.00 (reference)
II	95 (18.4)	44 (22.8)	0.052	1.58 (1.00–2.32)	83 (18.9)	39 (24.4)	0.056	1.62 (0.99–2.66)
III	34 (6.6)	18 (9.3)	0.008	2.66 (1.29–5.46)	26 (5.9)	15 (9.4)	0.006	3.10 (1.39–6.92)
IV	46 (8.9)	27 (14.0)	0.015	2.42 (1.19–4.95)	28 (6.3)	17 (10.6)	0.069	2.24 (0.94–5.36)
*P*_trend_				0.003				0.003
Tumor grade								
I	102 (19.7)	34 (17.6)		1.00 (reference)	89 (20.1)	28 (17.5)		1.00 (reference)
II	252 (48.8)	89 (46.1)	0.820	0.95 (0.59–1.53)	230 (52.0)	80 (50.0)	0.858	0.95 (0.57–1.61)
III	118 (22.8)	56 (29.0)	0.837	0.91 (0.46–1.79)	98 (22.2)	42 (26.3)	0.732	0.87 (0.40–1.91)
IV	45 (8.7)	14 (7.3)	0.309	0.43 (0.09–2.18)	25 (5.7)	10 (6.3)	0.889	0.85 (0.08–8.78)
*P*_trend_				0.451				0.283

*Adjusted for age, sex, BMI, smoking status, drinking status, diabetes and hypertension in logistic regression model. Analysis by stage was also adjusted for grade and grade for stage in addition to these variables. CI, confidence interval; OR, odds ratio.

## Discussion

In the present study, we evaluated the associations between the functional −31G>C polymorphism in the promoter of *survivin* and risk and progression of RCC. We found that individuals with the −31CC genotype had a significantly increased RCC risk of 1.50 and 1.38, compared with those with GG or GG/GC genotypes, respectively. The polymorphism was also associated with risk of developing advanced stage and moderately-differentiated RCC. Besides, we found that RCC patients carrying the CC genotype had a significantly greater prevalence of high clinical stage disease, compared with those with GG/GC genotypes. To the best of our knowledge, this is the first study to investigate the role of the *survivin* -31G>C polymorphism in the etiology of RCC.

As a unique inhibitor of apoptosis protein, survivin reduces the susceptibility of tumor cells to apoptotic stimuli and thereby promotes tumor cell survival during tumor development and progression [11], [12]. Survivin is strongly expressed in fetal tissues, but not expressed in adult normal tissues [27], [28]. However, over-expression of survivin was frequently observed in different types of cancer, including RCC. Lei *et al.* have demonstrated that the expression of survivin was elevated both in RCC cell lines and in tumor tissues [18]. Recently, several studies have suggested that the *survivin* promoter −31G>C polymorphism could modulate the expression of survivin [25], [29]. As mentioned before, this polymorphism is located at the CDE/CHR repressor binding site and may influence the affinity of repressor binding to this region, and then affect the expression of survivin. Functional studies have demonstrated that the −31C allele displayed a significantly higher transcriptional activity than the −31G allele, and individuals with the −31CC genotype had an increased survivin levels than those carrying the GC and GG genotypes [25]. Since survivin functions as an inhibitor of apoptosis protein that plays a crucial role in eliminating mutated or transformed cells from body, it is possible that individuals carrying the higher production genotype of *survivin* −31G>C polymorphism may possess a decreased apoptotic capacity to eliminate cells with DNA damage which may contribute to the occurrence of malignancies. Therefore, it is biologically plausible that the *survivin* promoter −31G>C polymorphism confers individuals susceptibility to RCC.

To date, a number of studies have attempted to evaluate the associations of the *survivin* −31G>C polymorphism and cancer risk [26]. The correlation between the *survivin* −31CC genotype and increased cancer risk has been identified by several other studies [26]. Although the role of this polymorphism in the etiology of RCC has not been assessed before, there are studies reporting that the *survivin* −31CC genotype was associated with increased risk of other urinary system cancer, such as urothelial carcinoma (OR = 4.0, 95%CI = 2.3–7.2) [30] and bladder cancer (OR = 1.85, 95%CI = 1.27–2.70) [31]. Our results are comparable to the results of these two studies and further suggest an important role of the *survivin* −31G>C polymorphism in the development of urinary system cancer. Most recently, a meta-analysis study has been conducted by Srivastava *et al.* to clarify the associations of this polymorphism and cancer risk [26]. They found that the *survivin* −31C allele was associated with 1.27 fold increased risk of cancer. The magnitude of RCC risk found with the C allele or CC genotype in the present study (OR = 1.11 and 1.38, respectively) were similar to the risk observed in the meta-analysis. However, the *survivin* −31G>C polymorphism was not identified as susceptibility locus for RCC by the recently published genome-wide association studies in European populations [3], [5]; and in our study, the p-value for the association between the polymorphism and RCC risk also did not meet genome-wide statistical significance. The *survivin* −31G>C polymorphism was not included in the GWAS conducted by Purdue *et al* [3], however, the GWAS results for a surrogate (rs3764384) in perfect with the −31G>C polymorphism was available. In their GWAS, the rs3764384 was not associated with RCC risk (*P* = 0.220, OR = 1.07, 95%CI = 0.96–1.18). According to the HapMap database, the rs3764384 is in complete linkage disequilibrium with the *survivin* −31G>C polymorphism in both Chinese Han populations and Caucasian populations (*D′* = 1, r^2^ = 1); the minor allele frequency of −31G>C (G allele) in Chinese Han population in Beijing is 0.477, which is dramatically different from that in European population (0.717). Therefore, we speculate that ethnicity differences might play a role in causing the disparity.

It has been proposed that expression level of *survivin* was a biomarker to predict RCC progression and prognosis [32]. Several studies have reported that elevated expression of survivin was associated with advanced tumor stage and grade, and patients with high survivin levels had a significantly shorter overall survival time than those with low survivin levels [20], [21], [22]. In the present study, we observed that that the *survivin* −31CC genotype was associated with progression of RCC, but these results should be interpreted cautiously since there is the possibility that the associations with poor prognosis are due to a late stage at diagnosis. Therefore, if confirmed by additional studies, this polymorphism may help to accurately predict the clinical course of RCC patients. RCC is characterized by high intra-tumor vascularity and, so far several anti-angiogenesis drugs (bevacizumab, pazopanib and sorafenib) have proven benefit in treating advanced RCC [33]. Since survivin has been suggested to promote tumor-associated angiogenesis and acts as a resistance factor to various anticancer therapies [34], [35], if individuals' drug susceptibility can be predicted by genetic variation such as the *survivin* −31G>C polymorphism, the efficacy of these drugs may be further enhanced. However, lack of available information on the drug treatment of our RCC patients and long-term follow-up restricts our study to explore the influence of the *survivin* −31G>C polymorphism on the efficacy of these drugs. Therefore, we urge that it be investigated in other studies which focus on the treatment of RCC patients.

When interpreting our results, another limitation should also be concerned. Because our case-control study was a hospital-based study, we could not rule out a possibility of selection bias of subjects that may have been associated with a particular genotype. However, the −31C allele frequency in our controls was similar to that in the HapMap database for Han Chinese in Beijing (46.4% vs. 48.8%) and was also similar to that in another population-based case-control study of Chinese population (46.4% vs. 47.6%). Besides, the genotype distributions of the polymorphism in our controls conformed to HWE. Therefore, the selection bias in terms of genotype distributions would not be substantial.

In conclusion, our case–control study indicates that the −31G>C polymorphism in the promoter of *survivin* have a significant influence on the occurrence and progression of RCC in Chinese population. Although the associations appeared to be statistically significant in our population, these initial findings should be independently verified by other large independent population-base studies. Besides, polymorphisms often vary between ethnic groups; therefore, additional studies are also required to clarify the association of this polymorphism with RCC risk in diverse ethnic populations.

## Supporting Information

Table S1 The sequences of the primers and probe used to genotype the survivin −31 G>C polymorphism.
(DOC)

Table S2 Stratification analyses between *Survivin* −31G>C genotypes and risk of RCC in cases and controls.
(DOC)
