# Supplementary material for: Functional Promoter -31G>C Variant in Survivin Gene Is Associated with Risk and Progression of Renal Cell Cancer in a Chinese Population
Source: PLoS One. 2012 Jan 25;7(1):e28829. doi: 10.1371/journal.pone.0028829 (PMC3266235; doi:10.1371/journal.pone.0028829)
Supplement: Table S1 — The sequences of the primers and probe used to genotype the survivin −31 G>C polymorphism. (DOC) [file pone.0028829.s001.doc]

Table S1 The sequences of the primers and probe used to genotype the survivin -31 G>C polymorphism

| Name | Sequence (5’-3’) |
| --- | --- |
| -31 G>C-F | CGTGCGCTCCCGACAT |
| -31 G>C-R | GATGCGGTGGTCCTTGAGAA |
| -31 G>C-G | FAM-TGAATCGCGGGACC-MGB |
| -31 G>C-C | HEX-TTGAATCGCCGGACC-MGB |
