# Supplementary material for: Functional Promoter -31G>C Variant in Survivin Gene Is Associated with Risk and Progression of Renal Cell Cancer in a Chinese Population
Source: PLoS One. 2012 Jan 25;7(1):e28829. doi: 10.1371/journal.pone.0028829 (PMC3266235; doi:10.1371/journal.pone.0028829)
Supplement: Table S2 — Stratification analyses between Survivin −31G>C genotypes and risk of RCC in cases and controls. (DOC) [file pone.0028829.s002.doc]

Table S2 Stratification analyses between *Survivin* -31G>C genotypes and risk of RCC in cases and controls

| Category | CG+GG | CC | *P* ^*^ | OR (95% CI) ^*^ | *P* _heterogeneity_ |
| --- | --- | --- | --- | --- | --- |
| Age |  |  |  |  | 0.453 |
| ≤57 | 262/337 | 102/86 | 0.031 | 1.45 (1.03-2.02) |  |
| >57 | 255/263 | 91/74 | 0.203 | 1.26 (0.88-1.81) |  |
| BMI^‡^ |  |  |  |  | 0.629 |
| <24 | 254/314 | 92/77 | 0.059 | 1.41 (0.99-2.00) |  |
| ≥24 | 263/286 | 101/83 | 0.102 | 1.33 (0.95-1.87) |  |
| Sex |  |  |  |  | 0.524 |
| Male | 334/384 | 120/106 | 0.119 | 1.27 (0.94-1.73) |  |
| Female | 183/216 | 73/54 | 0.038 | 1.54 (1.02-2.33) |  |
| Smoking status |  |  |  |  | 0.572 |
| Never | 325/404 | 119/111 | 0.086 | 1.30 (0.96-1.76) |  |
| Ever | 192/196 | 74/49 | 0.057 | 1.51 (0.99-2.32) |  |
| Smoking level (pack-years) |  |  |  |  | 0.207 |
| ≤20 | 462/537 | 171/149 | 0.053 | 1.29 (1.00-1.66) |  |
| >20 | 55/63 | 22/11 | 0.013 | 3.03 (1.26-7.28) |  |
| Drinking status |  |  |  |  | 0.119 |
| Never | 374/444 | 134/127 | 0.179 | 1.21 (0.92-1.61) |  |
| Ever | 143/156 | 59/33 | 0.009 | 1.95 (1.18-3.22) |  |

^*^ Adjusted for age, sex, BMI, pack-years of smoking, drinking status, hypertension and diabetes in logistic regression model; BMI, body mass index.
